# Supplementary material for: Design, development, and validation of multi-epitope proteins for serological diagnosis of Zika virus infections and discrimination from dengue virus seropositivity
Source: PLoS Negl Trop Dis. 2024 Apr 18;18(4):e0012100. doi: 10.1371/journal.pntd.0012100 (PMC11025737; doi:10.1371/journal.pntd.0012100)
Supplement: S1 Table — (DOCX) [file pntd.0012100.s001.docx]

| **Amino Acid sequence** | **Protein** | **Type of test used** | **Cross-reactivity assessment** | **Reference** |
| --- | --- | --- | --- | --- |
| SVEGELNAILEENGV | NS1 | Indirect ELISA | DENV e CHKV | (1) |
| WGKSYFVRAAKTNNSFVVDGDTLKECPLKH | NS1 | Indirect ELISA | DENV e CHKV | (1) |
| LVEDHGFGVFHTSVWLKVREDYSLECDPA | NS1 | Indirect ELISA | DENV e CHKV | (1) |
| PAVIGTAVKGKEAVH | NS1 | Indirect ELISA | DENV e CHKV | (1) |
| KNDTWRLKRAHLIEM | NS1 | Indirect ELISA | DENV e CHKV | (1) |
| IEESDLIIPKSLAGP | NS1 | Indirect ELISA | DENV e CHKV | (1) |
| SLAGPLSHHNTREGYRTQMKGPWHSEELEI | NS1 | Indirect ELISA | DENV e CHKV | (1) |
| GPWHSEELEIRFEEC | NS1 | Indirect ELISA | DENV e CHKV | (1) |
| DITWEKDAEVTGNSPRLDVA | NS2B | Indirect ELISA | DENV, CHKV, TBEV, JEV, WNV | (2) |
| HMCDATMSYECPMLDEGV | prM | ELISA | DENV | (3) |
| HKKGEARRSRRAVTLPSH | prM | ELISA | DENV | (3) |
| TVNMAEVRSYCYEASIS | E | ELISA | DENV | (3) |
| ISDMASDSRCTPTQGEAYL | E | ELISA | DENV | (3) |
| TGVFVYNDVEAWRDRYKY | NS1 | ELISA | DENV | (3) |
| REGYRTQMKGPWHSEELE | NS1 | ELISA | DENV | (3) |
| KRQTVVVLGS |  | Direct and sandwich ELISA | DENV | (4) |

References:

1 - Lee H-J, Cho Y, Kang HJ, Choi H, Han KR, Chong CK, et al. Identification of peptide based B-cell epitopes in Zika virus NS1. Biochem Biophys Res Commun. 2018 Nov;505(4):1010–4

2 - Virus Z, Elisa P, Elisa Z, Mishra N, Thakkar R, Ng J, et al. Chapter 10. 2020;2142:113–21.

3 - Kam Y-W, Leite JA, Amrun SN, Lum F-M, Yee W-X, Bakar FA, et al. ZIKV-Specific NS1 Epitopes as Serological Markers of Acute Zika Virus Infection. J Infect Dis. 2019 Jun;220(2):203–12.

4 - Kim DTH, Bao DT, Park H, Ngoc NM, Yeo SJ. Development of a novel peptide aptamer-based immunoassay to detect Zika virus in serum and urine. Theranostics. 2018;8(13):3629–42
