# Supplementary material for: Design, development, and validation of multi-epitope proteins for serological diagnosis of Zika virus infections and discrimination from dengue virus seropositivity
Source: PLoS Negl Trop Dis. 2024 Apr 18;18(4):e0012100. doi: 10.1371/journal.pntd.0012100 (PMC11025737; doi:10.1371/journal.pntd.0012100)
Supplement: S1 Fig — (A) Alignment of Epitope 1 with ten African strains and thirty-five Asian strains. (B) Alignment of Epitope 2 with ten African strains and thirty-five Asian strains. (C) Alignment of Epitope 3 with ten African strains and thirty-five Asian strains. (D) Alignment of Epitope 4 with ten African strains and thirty-five Asian strains. (E) Alignment of Epitope 5 with ten African strains and thirty-five Asian strains. (F) Alignment of Epitope 6 with ten African strains and thirty-five Asian strains. (G) Alignment of Epitope 7 with ten African strains and thirty-five Asian strains. (PDF) [file pntd.0012100.s002.pdf]

A

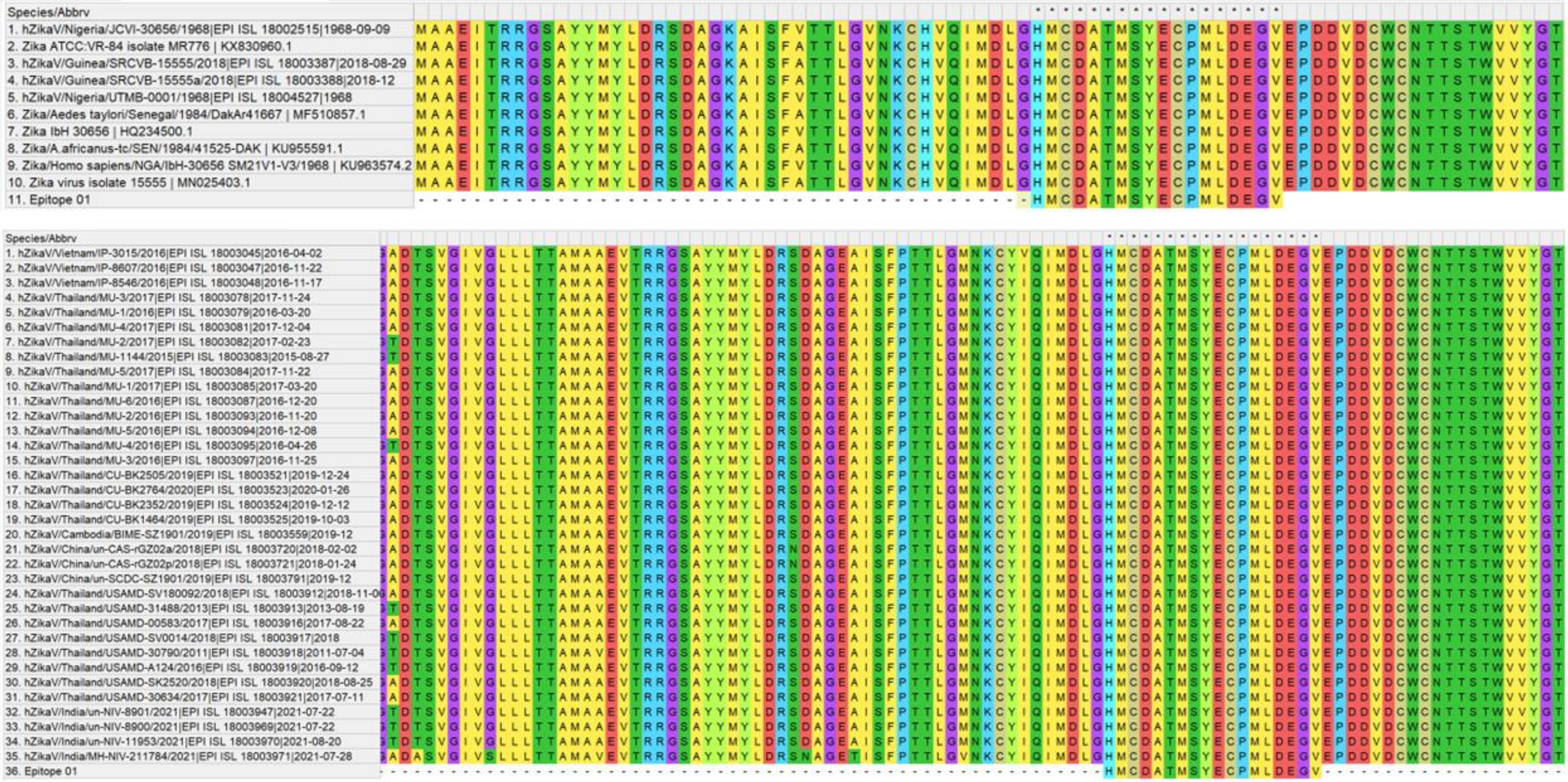
Species/Abbrv
1. hZikaV/Nigeria/JCVI-30656/1968|EPI ISL 18002515|1968-09-09
2. Zika ATCC:VR-84 isolate MR776 | KX830960.1
3. hZikaV/Guinea/SRCVB-15555/2018|EPI ISL 18003387|2018-08-29
4. hZikaV/Guinea/SRCVB-15555a/2018|EPI ISL 18003388|2018-12
5. hZikaV/Nigeria/UTMB-0001/1968|EPI ISL 18004527|1968
6. Zika/Aedes taylori/Senegal/1984/DakAr41667 | MF510857.1
7. Zika IbH 30656 | HQ234500.1
8. Zika/A.africanus-tc/SEN/1984/41525-DAK | KU955591.1
9. Zika/Homo sapiens/NGA/IbH-30656 SM21V1-V3/1968 | KU963574.2
10. Zika virus isolate 15555 | MN025403.1
11. Epitope 01
HMCDATMSYECPMLDEGV
Species/Abbrv
1. hZikaV/Vietnam/IP-3015/2016|EPI ISL 18003045|2016-04-02
2. hZikaV/Vietnam/IP-8607/2016|EPI ISL 18003047|2016-11-22
3. hZikaV/Vietnam/IP-8546/2016|EPI ISL 18003048|2016-11-17
4. hZikaV/Thailand/MU-3/2017|EPI ISL 18003078|2017-11-24
5. hZikaV/Thailand/MU-1/2016|EPI ISL 18003079|2016-03-20
6. hZikaV/Thailand/MU-4/2017|EPI ISL 18003081|2017-12-04
7. hZikaV/Thailand/MU-2/2017|EPI ISL 18003082|2017-02-23
8. hZikaV/Thailand/MU-1144/2015|EPI ISL 18003083|2015-08-27
9. hZikaV/Thailand/MU-5/2017|EPI ISL 18003084|2017-11-22
10. hZikaV/Thailand/MU-1/2017|EPI ISL 18003085|2017-03-20
11. hZikaV/Thailand/MU-6/2016|EPI ISL 18003087|2016-12-20
12. hZikaV/Thailand/MU-2/2016|EPI ISL 18003093|2016-11-20
13. hZikaV/Thailand/MU-5/2016|EPI ISL 18003094|2016-12-08
14. hZikaV/Thailand/MU-4/2016|EPI ISL 18003095|2016-04-26
15. hZikaV/Thailand/MU-3/2016|EPI ISL 18003097|2016-11-25
16. hZikaV/Thailand/CU-BK2505/2019|EPI ISL 18003521|2019-12-24
17. hZikaV/Thailand/CU-BK2764/2020|EPI ISL 18003523|2020-01-26
18. hZikaV/Thailand/CU-BK2352/2019|EPI ISL 18003524|2019-12-12
19. hZikaV/Thailand/CU-BK1464/2019|EPI ISL 18003525|2019-10-03
20. hZikaV/Cambodia/BIME-SZ1901/2019|EPI ISL 18003559|2019-12
21. hZikaV/China/un-CAS-rGZ02a/2018|EPI ISL 18003720|2018-02-02
22. hZikaV/China/un-CAS-rGZ02p/2018|EPI ISL 18003721|2018-01-24
23. hZikaV/China/un-SCDC-SZ1901/2019|EPI ISL 18003791|2019-12
24. hZikaV/Thailand/USAMD-SV180092/2018|EPI ISL 18003912|2018-11-06
25. hZikaV/Thailand/USAMD-31488/2013|EPI ISL 18003913|2013-08-19
26. hZikaV/Thailand/USAMD-00583/2017|EPI ISL 18003916|2017-08-22
27. hZikaV/Thailand/USAMD-SV0014/2018|EPI ISL 18003917|2018
28. hZikaV/Thailand/USAMD-30790/2011|EPI ISL 18003918|2011-07-04
29. hZikaV/Thailand/USAMD-A124/2016|EPI ISL 18003919|2016-09-12
30. hZikaV/Thailand/USAMD-SK2520/2018|EPI ISL 18003920|2018-08-25
31. hZikaV/Thailand/USAMD-30634/2017|EPI ISL 18003921|2017-07-11
32. hZikaV/India/un-NIV-8901/2021|EPI ISL 18003947|2021-07-22
33. hZikaV/India/un-NIV-8900/2021|EPI ISL 18003969|2021-07-22
34. hZikaV/India/un-NIV-11953/2021|EPI ISL 18003970|2021-08-20
35. hZikaV/India/MH-NIV-211784/2021|EPI ISL 18003971|2021-07-28
36. Epitope 01
HMCDATMSYECPMLDEGV

B

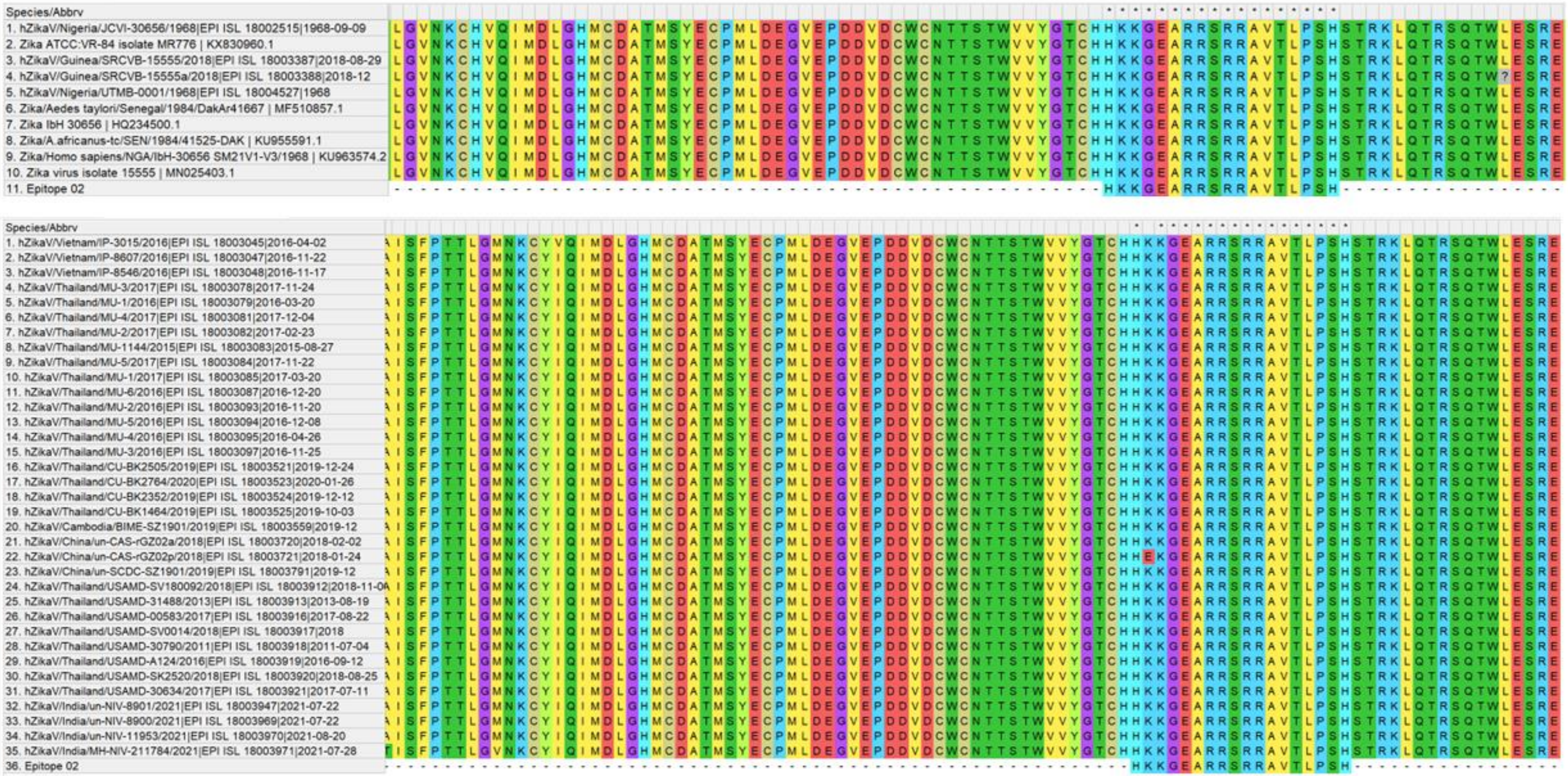
Species/Abbrv
1. hZikaV/Nigeria/JCVI-30656/1968|EPI ISL 18002515|1968-09-09
2. Zika ATCC:VR-84 isolate MR776 | KX830960.1
3. hZikaV/Guinea/SRCVB-15555/2018|EPI ISL 18003387|2018-08-29
4. hZikaV/Guinea/SRCVB-15555a/2018|EPI ISL 18003388|2018-12
5. hZikaV/Nigeria/UTMB-0001/1968|EPI ISL 18004527|1968
6. Zika/Aedes taylori/Senegal/1984/DakAr41667 | MF510857.1
7. Zika IbH 30656 | HQ234500.1
8. Zika/A.africanus-tc/SEN/1984/41525-DAK | KU955591.1
9. Zika/Homo sapiens/NGA/IbH-30656 SM21V1-V3/1968 | KU963574.2
10. Zika virus isolate 15555 | MN025403.1
11. Epitope 02
HKKGEARRSRRAVTLPSH
Species/Abbrv
1. hZikaV/Vietnam/IP-3015/2016|EPI ISL 18003045|2016-04-02
2. hZikaV/Vietnam/IP-8607/2016|EPI ISL 18003047|2016-11-22
3. hZikaV/Vietnam/IP-8546/2016|EPI ISL 18003048|2016-11-17
4. hZikaV/Thailand/MU-3/2017|EPI ISL 18003078|2017-11-24
5. hZikaV/Thailand/MU-1/2016|EPI ISL 18003079|2016-03-20
6. hZikaV/Thailand/MU-4/2017|EPI ISL 18003081|2017-12-04
7. hZikaV/Thailand/MU-2/2017|EPI ISL 18003082|2017-02-23
8. hZikaV/Thailand/MU-1144/2015|EPI ISL 18003083|2015-08-27
9. hZikaV/Thailand/MU-5/2017|EPI ISL 18003084|2017-11-22
10. hZikaV/Thailand/MU-1/2017|EPI ISL 18003085|2017-03-20
11. hZikaV/Thailand/MU-6/2016|EPI ISL 18003087|2016-12-20
12. hZikaV/Thailand/MU-2/2016|EPI ISL 18003093|2016-11-20
13. hZikaV/Thailand/MU-5/2016|EPI ISL 18003094|2016-12-08
14. hZikaV/Thailand/MU-4/2016|EPI ISL 18003095|2016-04-26
15. hZikaV/Thailand/MU-3/2016|EPI ISL 18003097|2016-11-25
16. hZikaV/Thailand/CU-BK2505/2019|EPI ISL 18003521|2019-12-24
17. hZikaV/Thailand/CU-BK2764/2020|EPI ISL 18003523|2020-01-26
18. hZikaV/Thailand/CU-BK2352/2019|EPI ISL 18003524|2019-12-12
19. hZikaV/Thailand/CU-BK1464/2019|EPI ISL 18003525|2019-10-03
20. hZikaV/Cambodia/BIME-SZ1901/2019|EPI ISL 18003559|2019-12
21. hZikaV/China/un-CAS-rGZ02a/2018|EPI ISL 18003720|2018-02-02
22. hZikaV/China/un-CAS-rGZ02p/2018|EPI ISL 18003721|2018-01-24
23. hZikaV/China/un-SCDC-SZ1901/2019|EPI ISL 18003791|2019-12
24. hZikaV/Thailand/USAMD-SV180092/2018|EPI ISL 18003912|2018-11-0
25. hZikaV/Thailand/USAMD-31488/2013|EPI ISL 18003913|2013-08-19
26. hZikaV/Thailand/USAMD-00583/2017|EPI ISL 18003916|2017-08-22
27. hZikaV/Thailand/USAMD-SV0014/2018|EPI ISL 18003917|2018
28. hZikaV/Thailand/USAMD-30790/2011|EPI ISL 18003918|2011-07-04
29. hZikaV/Thailand/USAMD-A124/2016|EPI ISL 18003919|2016-09-12
30. hZikaV/Thailand/USAMD-SK2520/2018|EPI ISL 18003920|2018-08-25
31. hZikaV/Thailand/USAMD-30634/2017|EPI ISL 18003921|2017-07-11
32. hZikaV/India/un-NIV-8901/2021|EPI ISL 18003947|2021-07-22
33. hZikaV/India/un-NIV-8900/2021|EPI ISL 18003969|2021-07-22
34. hZikaV/India/un-NIV-11953/2021|EPI ISL 18003970|2021-08-20
35. hZikaV/India/MH-NIV-211784/2021|EPI ISL 18003971|2021-07-28
36. Epitope 02
HKKGEARRSRRAVTLPSH

C

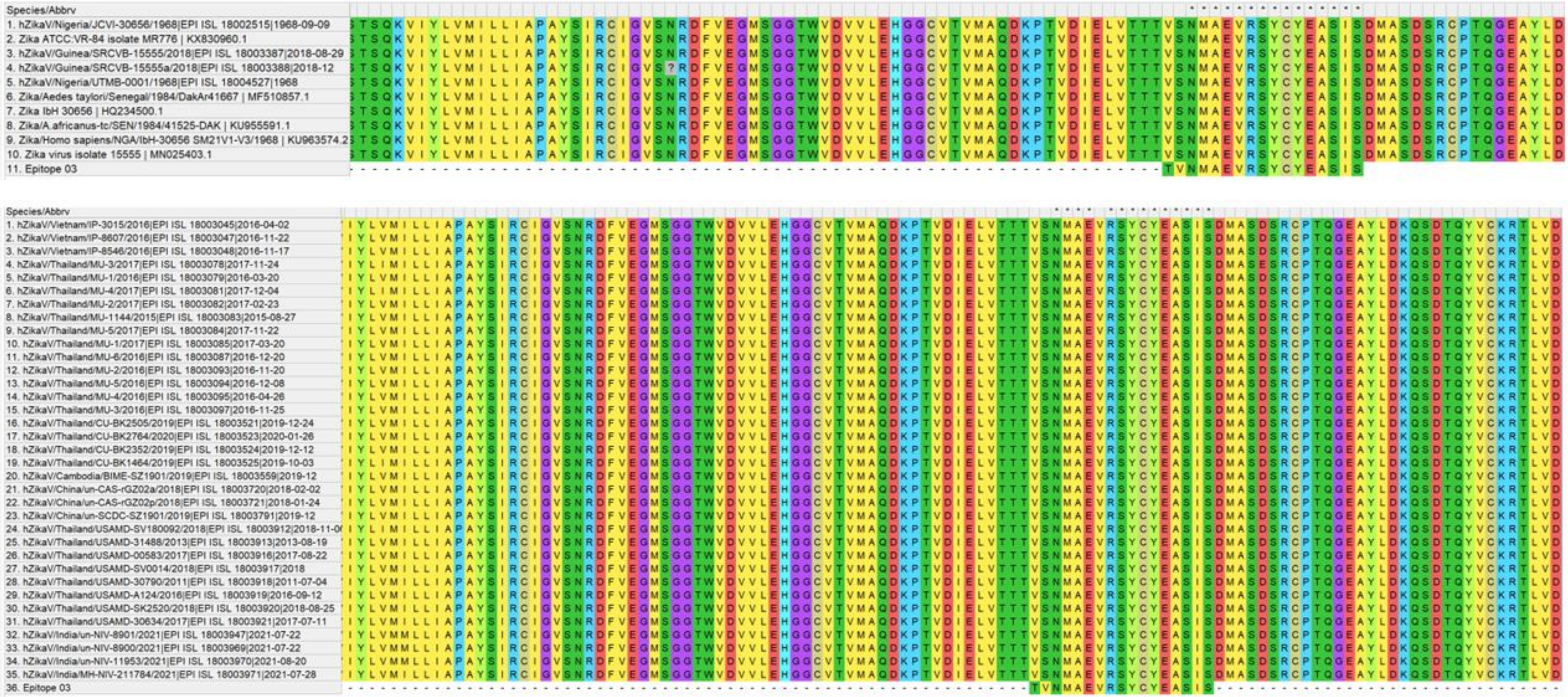
Species/Abbrv
1. hZikaV/Nigeria/JCVI-30656/1968|EPI ISL 18002515|1968-09-09
2. Zika ATCC:VR-84 isolate MR776 | KX830960.1
3. hZikaV/Guinea/SRCVB-15555/2018|EPI ISL 18003387|2018-08-29
4. hZikaV/Guinea/SRCVB-15555a/2018|EPI ISL 18003388|2018-12
5. hZikaV/Nigeria/UTMB-0001/1968|EPI ISL 18004527|1968
6. Zika/Aedes taylori/Senegal/1984/DakAr41667 | MF510857.1
7. Zika IbH 30656 | HQ234500.1
8. Zika/A.africanus-tc/SEN/1984/41525-DAK | KU955591.1
9. Zika/Homo sapiens/NGA/IbH-30656 SM21V1-V3/1968 | KU963574.2
10. Zika virus isolate 15555 | MN025403.1
11. Epitope 03
TVNMAEVRSYCYEASIS
Species/Abbrv
1. hZikaV/Vietnam/IP-3015/2016|EPI ISL 18003045|2016-04-02
2. hZikaV/Vietnam/IP-8607/2016|EPI ISL 18003047|2016-11-22
3. hZikaV/Vietnam/IP-8546/2016|EPI ISL 18003048|2016-11-17
4. hZikaV/Thailand/MU-3/2017|EPI ISL 18003078|2017-11-24
5. hZikaV/Thailand/MU-1/2016|EPI ISL 18003079|2016-03-20
6. hZikaV/Thailand/MU-4/2017|EPI ISL 18003081|2017-12-04
7. hZikaV/Thailand/MU-2/2017|EPI ISL 18003082|2017-02-23
8. hZikaV/Thailand/MU-1144/2015|EPI ISL 18003083|2015-08-27
9. hZikaV/Thailand/MU-5/2017|EPI ISL 18003084|2017-11-22
10. hZikaV/Thailand/MU-1/2017|EPI ISL 18003085|2017-03-20
11. hZikaV/Thailand/MU-6/2016|EPI ISL 18003087|2016-12-20
12. hZikaV/Thailand/MU-2/2016|EPI ISL 18003093|2016-11-20
13. hZikaV/Thailand/MU-5/2016|EPI ISL 18003094|2016-12-08
14. hZikaV/Thailand/MU-4/2016|EPI ISL 18003095|2016-04-26
15. hZikaV/Thailand/MU-3/2016|EPI ISL 18003097|2016-11-25
16. hZikaV/Thailand/CU-BK2505/2019|EPI ISL 18003521|2019-12-24
17. hZikaV/Thailand/CU-BK2764/2020|EPI ISL 18003523|2020-01-26
18. hZikaV/Thailand/CU-BK2352/2019|EPI ISL 18003524|2019-12-12
19. hZikaV/Thailand/CU-BK1464/2019|EPI ISL 18003525|2019-10-03
20. hZikaV/Cambodia/BIME-SZ1901/2019|EPI ISL 18003559|2019-12
21. hZikaV/China/un-CAS-rGZ02a/2018|EPI ISL 18003720|2018-02-02
22. hZikaV/China/un-CAS-rGZ02p/2018|EPI ISL 18003721|2018-01-24
23. hZikaV/China/un-SCDC-SZ1901/2019|EPI ISL 18003791|2019-12
24. hZikaV/Thailand/USAMD-SV180092/2018|EPI ISL 18003912|2018-11-0
25. hZikaV/Thailand/USAMD-31488/2013|EPI ISL 18003913|2013-08-19
26. hZikaV/Thailand/USAMD-00583/2017|EPI ISL 18003916|2017-08-22
27. hZikaV/Thailand/USAMD-SV0014/2018|EPI ISL 18003917|2018
28. hZikaV/Thailand/USAMD-30790/2011|EPI ISL 18003918|2011-07-04
29. hZikaV/Thailand/USAMD-A124/2016|EPI ISL 18003919|2016-09-12
30. hZikaV/Thailand/USAMD-SK2520/2018|EPI ISL 18003920|2018-08-25
31. hZikaV/Thailand/USAMD-30634/2017|EPI ISL 18003921|2017-07-11
32. hZikaV/India/un-NIV-8901/2021|EPI ISL 18003947|2021-07-22
33. hZikaV/India/un-NIV-8900/2021|EPI ISL 18003969|2021-07-22
34. hZikaV/India/un-NIV-11953/2021|EPI ISL 18003970|2021-08-20
35. hZikaV/India/MH-NIV-211784/2021|EPI ISL 18003971|2021-07-28
36. Epitope 03
TVNMAEVRSYCYEASIS

D

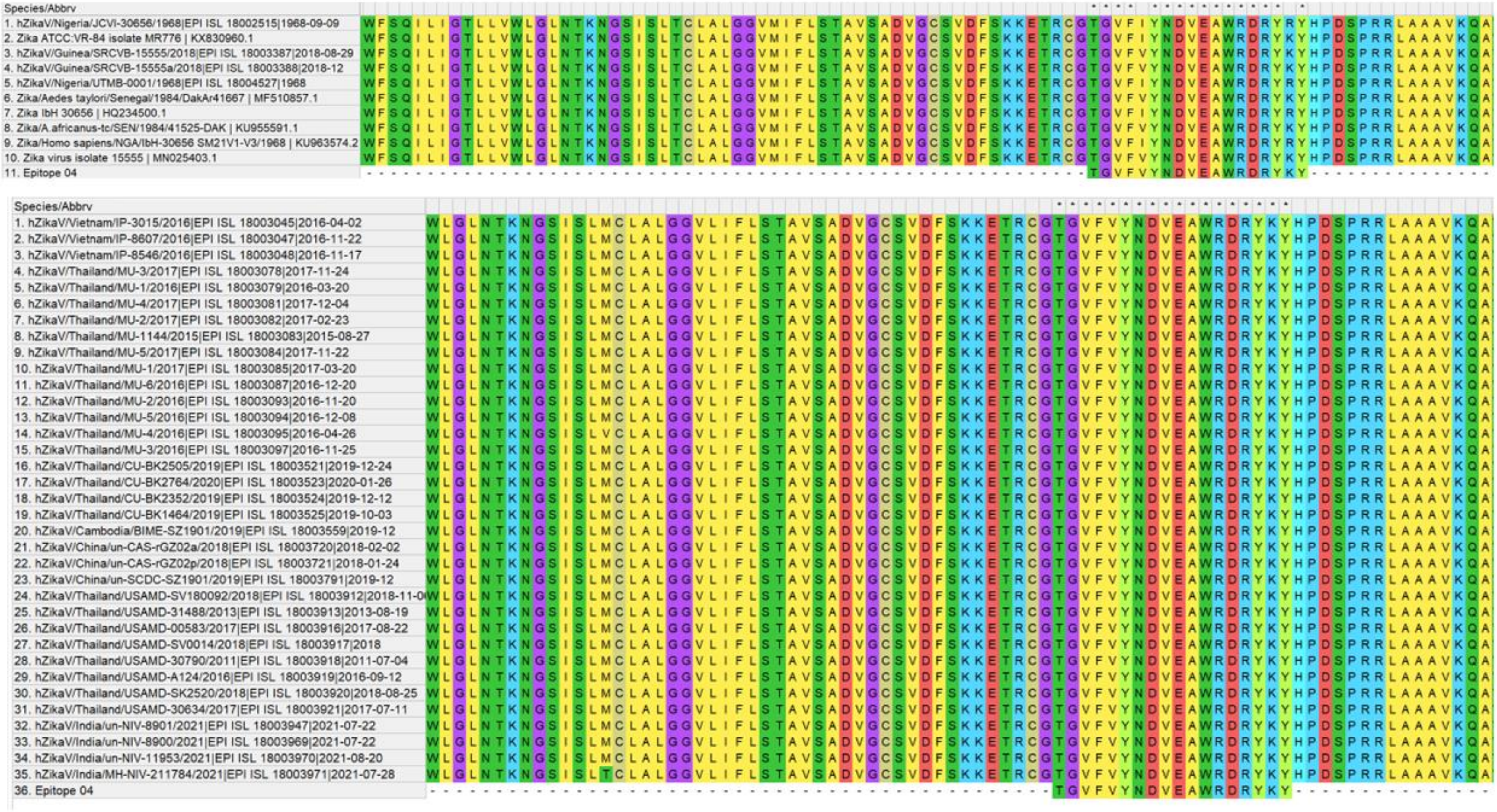

Species/Abbrv
1. hZikaV/Nigeria/JCVI-30656/1968|EPI ISL 18002515|1968-09-09
2. Zika ATCC:VR-84 isolate MR776 | KX830960.1
3. hZikaV/Guinea/SRCVB-15555/2018|EPI ISL 18003387|2018-08-29
4. hZikaV/Guinea/SRCVB-15555a/2018|EPI ISL 18003388|2018-12
5. hZikaV/Nigeria/UTMB-0001/1968|EPI ISL 18004527|1968
6. Zika/Aedes taylori/Senegal/1984/DakAr41667 | MF510857.1
7. Zika IbH 30656 | HQ234500.1
8. Zika/A.africanus-tc/SEN/1984/41525-DAK | KU955591.1
9. Zika/Homo sapiens/NGA/IbH-30656 SM21V1-V3/1968 | KU963574.2
10. Zika virus isolate 15555 | MN025403.1
11. Epitope 04
TGVFVYNDVEAWRDRYKY
Species/Abbrv
1. hZikaV/Vietnam/IP-3015/2016|EPI ISL 18003045|2016-04-02
2. hZikaV/Vietnam/IP-8607/2016|EPI ISL 18003047|2016-11-22
3. hZikaV/Vietnam/IP-8546/2016|EPI ISL 18003048|2016-11-17
4. hZikaV/Thailand/MU-3/2017|EPI ISL 18003078|2017-11-24
5. hZikaV/Thailand/MU-1/2016|EPI ISL 18003079|2016-03-20
6. hZikaV/Thailand/MU-4/2017|EPI ISL 18003081|2017-12-04
7. hZikaV/Thailand/MU-2/2017|EPI ISL 18003082|2017-02-23
8. hZikaV/Thailand/MU-1144/2015|EPI ISL 18003083|2015-08-27
9. hZikaV/Thailand/MU-5/2017|EPI ISL 18003084|2017-11-22
10. hZikaV/Thailand/MU-1/2017|EPI ISL 18003085|2017-03-20
11. hZikaV/Thailand/MU-6/2016|EPI ISL 18003087|2016-12-20
12. hZikaV/Thailand/MU-2/2016|EPI ISL 18003093|2016-11-20
13. hZikaV/Thailand/MU-5/2016|EPI ISL 18003094|2016-12-08
14. hZikaV/Thailand/MU-4/2016|EPI ISL 18003095|2016-04-26
15. hZikaV/Thailand/MU-3/2016|EPI ISL 18003097|2016-11-25
16. hZikaV/Thailand/CU-BK2505/2019|EPI ISL 18003521|2019-12-24
17. hZikaV/Thailand/CU-BK2764/2020|EPI ISL 18003523|2020-01-26
18. hZikaV/Thailand/CU-BK2352/2019|EPI ISL 18003524|2019-12-12
19. hZikaV/Thailand/CU-BK1464/2019|EPI ISL 18003525|2019-10-03
20. hZikaV/Cambodia/BIME-SZ1901/2019|EPI ISL 18003559|2019-12
21. hZikaV/China/un-CAS-rGZ02a/2018|EPI ISL 18003720|2018-02-02
22. hZikaV/China/un-CAS-rGZ02p/2018|EPI ISL 18003721|2018-01-24
23. hZikaV/China/un-SCDC-SZ1901/2019|EPI ISL 18003791|2019-12
24. hZikaV/Thailand/USAMD-SV180092/2018|EPI ISL 18003912|2018-11-0
25. hZikaV/Thailand/USAMD-31488/2013|EPI ISL 18003913|2013-08-19
26. hZikaV/Thailand/USAMD-00583/2017|EPI ISL 18003916|2017-08-22
27. hZikaV/Thailand/USAMD-SV0014/2018|EPI ISL 18003917|2018
28. hZikaV/Thailand/USAMD-30790/2011|EPI ISL 18003918|2011-07-04
29. hZikaV/Thailand/USAMD-A124/2016|EPI ISL 18003919|2016-09-12
30. hZikaV/Thailand/USAMD-SK2520/2018|EPI ISL 18003920|2018-08-25
31. hZikaV/Thailand/USAMD-30634/2017|EPI ISL 18003921|2017-07-11
32. hZikaV/India/un-NIV-8901/2021|EPI ISL 18003947|2021-07-22
33. hZikaV/India/un-NIV-8900/2021|EPI ISL 18003969|2021-07-22
34. hZikaV/India/un-NIV-11953/2021|EPI ISL 18003970|2021-08-20
35. hZikaV/India/MH-NIV-211784/2021|EPI ISL 18003971|2021-07-28
36. Epitope 04
TGVFVYNDVEAWRDRYKY

E

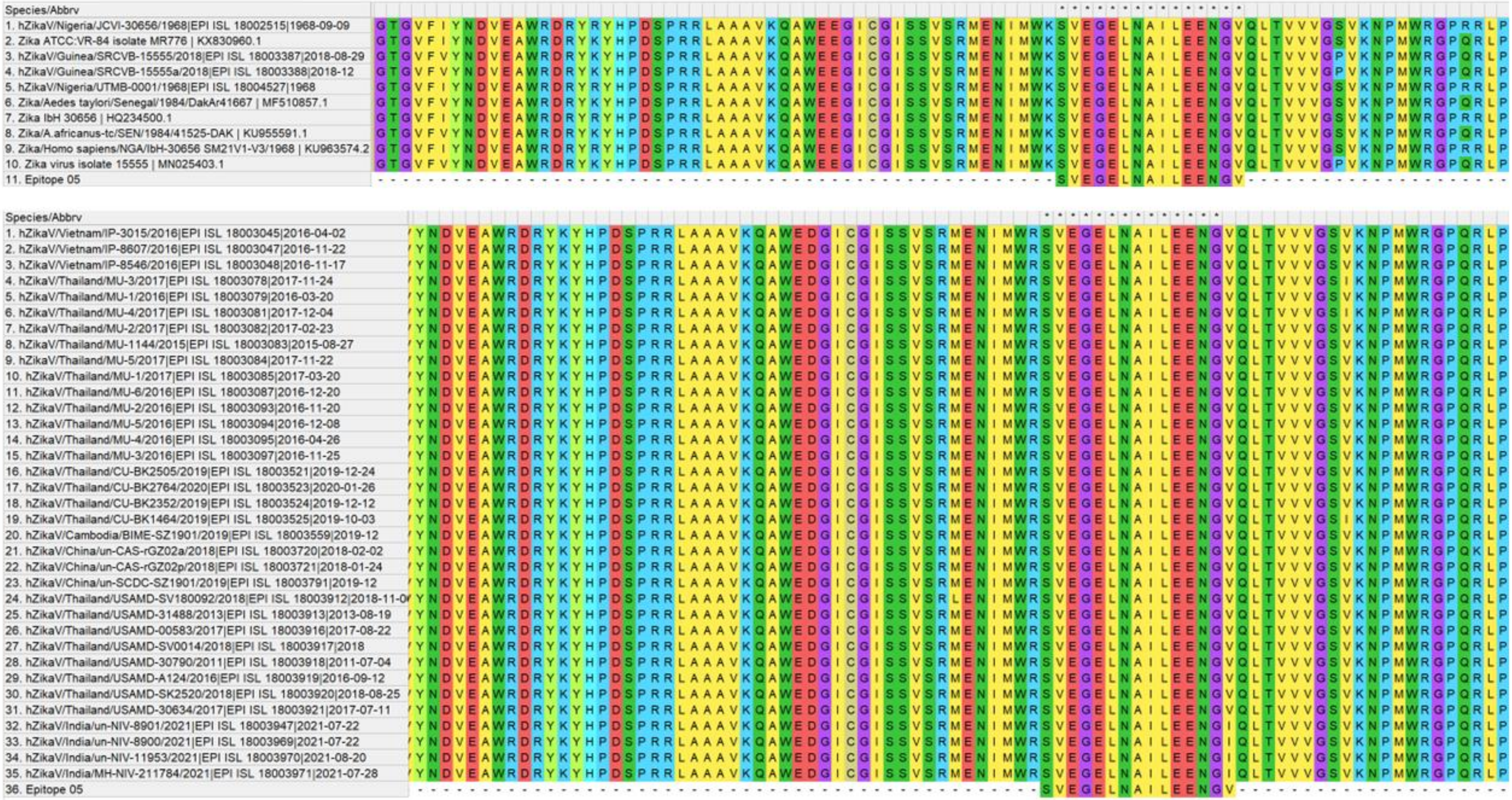

Species/Abbrv
1. hZikaV/Nigeria/JCVI-30656/1968|EPI ISL 18002515|1968-09-09
2. Zika ATCC:VR-84 isolate MR776 | KX830960.1
3. hZikaV/Guinea/SRCVB-15555/2018|EPI ISL 18003387|2018-08-29
4. hZikaV/Guinea/SRCVB-15555a/2018|EPI ISL 18003388|2018-12
5. hZikaV/Nigeria/UTMB-0001/1968|EPI ISL 18004527|1968
6. Zika/Aedes taylori/Senegal/1984/DakAr41667 | MF510857.1
7. Zika IbH 30656 | HQ234500.1
8. Zika/A.africanus-tc/SEN/1984/41525-DAK | KU955591.1
9. Zika/Homo sapiens/NGA/IbH-30656 SM21V1-V3/1968 | KU963574.2
10. Zika virus isolate 15555 | MN025403.1
11. Epitope 05
SVEGELNAILEENGV
Species/Abbrv
1. hZikaV/Vietnam/IP-3015/2016|EPI ISL 18003045|2016-04-02
2. hZikaV/Vietnam/IP-8607/2016|EPI ISL 18003047|2016-11-22
3. hZikaV/Vietnam/IP-8546/2016|EPI ISL 18003048|2016-11-17
4. hZikaV/Thailand/MU-3/2017|EPI ISL 18003078|2017-11-24
5. hZikaV/Thailand/MU-1/2016|EPI ISL 18003079|2016-03-20
6. hZikaV/Thailand/MU-4/2017|EPI ISL 18003081|2017-12-04
7. hZikaV/Thailand/MU-2/2017|EPI ISL 18003082|2017-02-23
8. hZikaV/Thailand/MU-1144/2015|EPI ISL 18003083|2015-08-27
9. hZikaV/Thailand/MU-5/2017|EPI ISL 18003084|2017-11-22
10. hZikaV/Thailand/MU-1/2017|EPI ISL 18003085|2017-03-20
11. hZikaV/Thailand/MU-6/2016|EPI ISL 18003087|2016-12-20
12. hZikaV/Thailand/MU-2/2016|EPI ISL 18003093|2016-11-20
13. hZikaV/Thailand/MU-5/2016|EPI ISL 18003094|2016-12-08
14. hZikaV/Thailand/MU-4/2016|EPI ISL 18003095|2016-04-26
15. hZikaV/Thailand/MU-3/2016|EPI ISL 18003097|2016-11-25
16. hZikaV/Thailand/CU-BK2505/2019|EPI ISL 18003521|2019-12-24
17. hZikaV/Thailand/CU-BK2764/2020|EPI ISL 18003523|2020-01-26
18. hZikaV/Thailand/CU-BK2352/2019|EPI ISL 18003524|2019-12-12
19. hZikaV/Thailand/CU-BK1464/2019|EPI ISL 18003525|2019-10-03
20. hZikaV/Cambodia/BIME-SZ1901/2019|EPI ISL 18003559|2019-12
21. hZikaV/China/un-CAS-rGZ02a/2018|EPI ISL 18003720|2018-02-02
22. hZikaV/China/un-CAS-rGZ02p/2018|EPI ISL 18003721|2018-01-24
23. hZikaV/China/un-SCDC-SZ1901/2019|EPI ISL 18003791|2019-12
24. hZikaV/Thailand/USAMD-SV180092/2018|EPI ISL 18003912|2018-11-0
25. hZikaV/Thailand/USAMD-31488/2013|EPI ISL 18003913|2013-08-19
26. hZikaV/Thailand/USAMD-00583/2017|EPI ISL 18003916|2017-08-22
27. hZikaV/Thailand/USAMD-SV0014/2018|EPI ISL 18003917|2018
28. hZikaV/Thailand/USAMD-30790/2011|EPI ISL 18003918|2011-07-04
29. hZikaV/Thailand/USAMD-A124/2016|EPI ISL 18003919|2016-09-12
30. hZikaV/Thailand/USAMD-SK2520/2018|EPI ISL 18003920|2018-08-25
31. hZikaV/Thailand/USAMD-30634/2017|EPI ISL 18003921|2017-07-11
32. hZikaV/India/un-NIV-8901/2021|EPI ISL 18003947|2021-07-22
33. hZikaV/India/un-NIV-8900/2021|EPI ISL 18003969|2021-07-22
34. hZikaV/India/un-NIV-11953/2021|EPI ISL 18003970|2021-08-20
35. hZikaV/India/MH-NIV-211784/2021|EPI ISL 18003971|2021-07-28
36. Epitope 05
SVEGELNAILEENGV

F

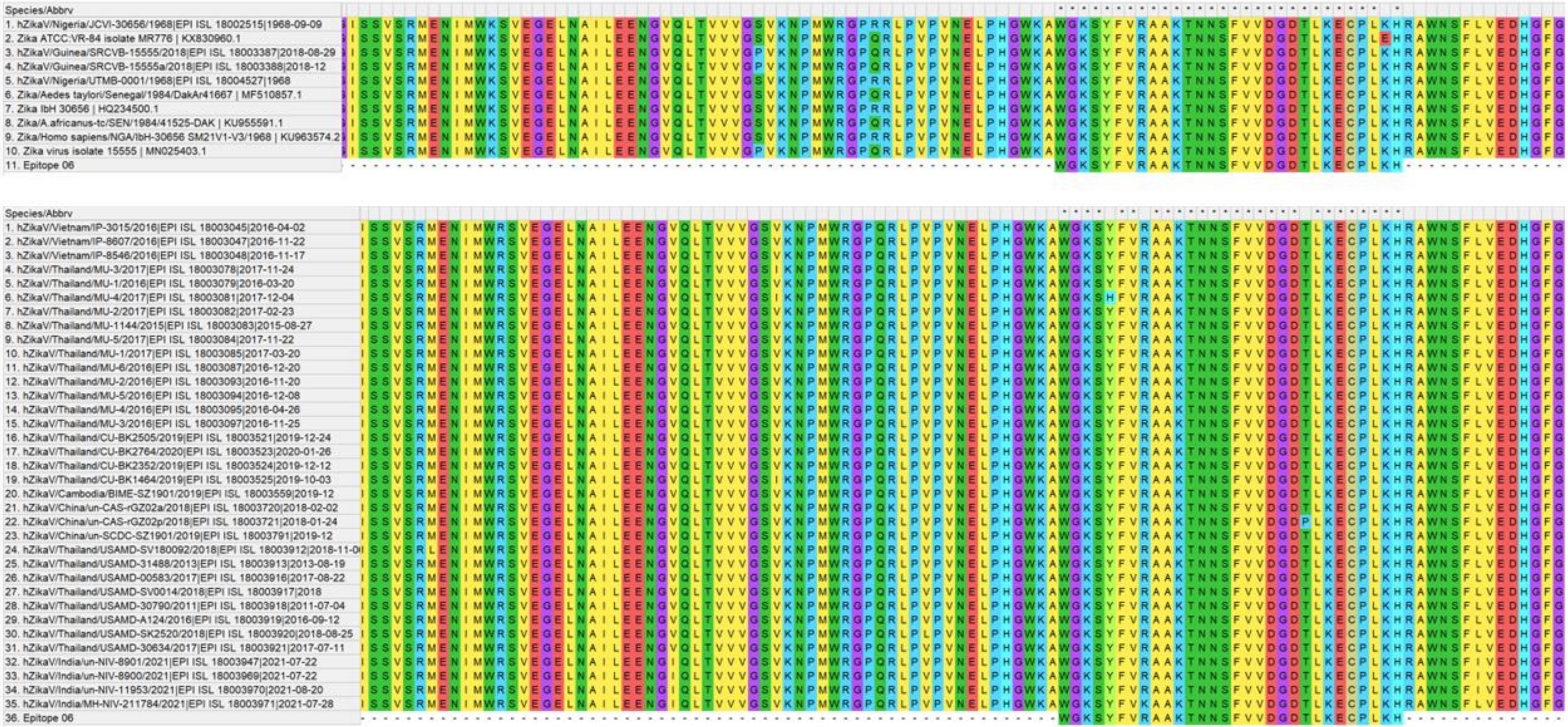
Species/Abbrv
1. hZikaV/Nigeria/JCVI-30656/1968|EPI ISL 18002515|1968-09-09
2. Zika ATCC:VR-84 isolate MR776 | KX830960.1
3. hZikaV/Guinea/SRCVB-15555/2018|EPI ISL 18003387|2018-08-29
4. hZikaV/Guinea/SRCVB-15555a/2018|EPI ISL 18003388|2018-12
5. hZikaV/Nigeria/UTMB-0001/1968|EPI ISL 18004527|1968
6. Zika/Aedes taylori/Senegal/1984/DakAr41667 | MF510857.1
7. Zika IbH 30656 | HQ234500.1
8. Zika/A.africanus-tc/SEN/1984/41525-DAK | KU955591.1
9. Zika/Homo sapiens/NGA/IbH-30656 SM21V1-V3/1968 | KU963574.2
10. Zika virus isolate 15555 | MN025403.1
11. Epitope 06
WGKSYFVRAAKTNNSFVVDGDTLKECPLKH
Species/Abbrv
1. hZikaV/Vietnam/IP-3015/2016|EPI ISL 18003045|2016-04-02
2. hZikaV/Vietnam/IP-8607/2016|EPI ISL 18003047|2016-11-22
3. hZikaV/Vietnam/IP-8546/2016|EPI ISL 18003048|2016-11-17
4. hZikaV/Thailand/MU-3/2017|EPI ISL 18003078|2017-11-24
5. hZikaV/Thailand/MU-1/2016|EPI ISL 18003079|2016-03-20
6. hZikaV/Thailand/MU-4/2017|EPI ISL 18003081|2017-12-04
7. hZikaV/Thailand/MU-2/2017|EPI ISL 18003082|2017-02-23
8. hZikaV/Thailand/MU-1144/2015|EPI ISL 18003083|2015-08-27
9. hZikaV/Thailand/MU-5/2017|EPI ISL 18003084|2017-11-22
10. hZikaV/Thailand/MU-1/2017|EPI ISL 18003085|2017-03-20
11. hZikaV/Thailand/MU-6/2016|EPI ISL 18003087|2016-12-20
12. hZikaV/Thailand/MU-2/2016|EPI ISL 18003093|2016-11-20
13. hZikaV/Thailand/MU-5/2016|EPI ISL 18003094|2016-12-08
14. hZikaV/Thailand/MU-4/2016|EPI ISL 18003095|2016-04-26
15. hZikaV/Thailand/MU-3/2016|EPI ISL 18003097|2016-11-25
16. hZikaV/Thailand/CU-BK2505/2019|EPI ISL 18003521|2019-12-24
17. hZikaV/Thailand/CU-BK2764/2020|EPI ISL 18003523|2020-01-26
18. hZikaV/Thailand/CU-BK2352/2019|EPI ISL 18003524|2019-12-12
19. hZikaV/Thailand/CU-BK1464/2019|EPI ISL 18003525|2019-10-03
20. hZikaV/Cambodia/BIME-SZ1901/2019|EPI ISL 18003559|2019-12
21. hZikaV/China/un-CAS-rGZ02a/2018|EPI ISL 18003720|2018-02-02
22. hZikaV/China/un-CAS-rGZ02p/2018|EPI ISL 18003721|2018-01-24
23. hZikaV/China/un-SCDC-SZ1901/2019|EPI ISL 18003791|2019-12
24. hZikaV/Thailand/USAMD-SV180092/2018|EPI ISL 18003912|2018-11-0
25. hZikaV/Thailand/USAMD-31488/2013|EPI ISL 18003913|2013-08-19
26. hZikaV/Thailand/USAMD-00583/2017|EPI ISL 18003916|2017-08-22
27. hZikaV/Thailand/USAMD-SV0014/2018|EPI ISL 18003917|2018
28. hZikaV/Thailand/USAMD-30790/2011|EPI ISL 18003918|2011-07-04
29. hZikaV/Thailand/USAMD-A124/2016|EPI ISL 18003919|2016-09-12
30. hZikaV/Thailand/USAMD-SK2520/2018|EPI ISL 18003920|2018-08-25
31. hZikaV/Thailand/USAMD-30634/2017|EPI ISL 18003921|2017-07-11
32. hZikaV/India/un-NIV-8901/2021|EPI ISL 18003947|2021-07-22
33. hZikaV/India/un-NIV-8900/2021|EPI ISL 18003969|2021-07-22
34. hZikaV/India/un-NIV-11953/2021|EPI ISL 18003970|2021-08-20
35. hZikaV/India/MH-NIV-211784/2021|EPI ISL 18003971|2021-07-28
36. Epitope 06
WGKSYFVRAAKTNNSFVVDGDTLKECPLKH

G

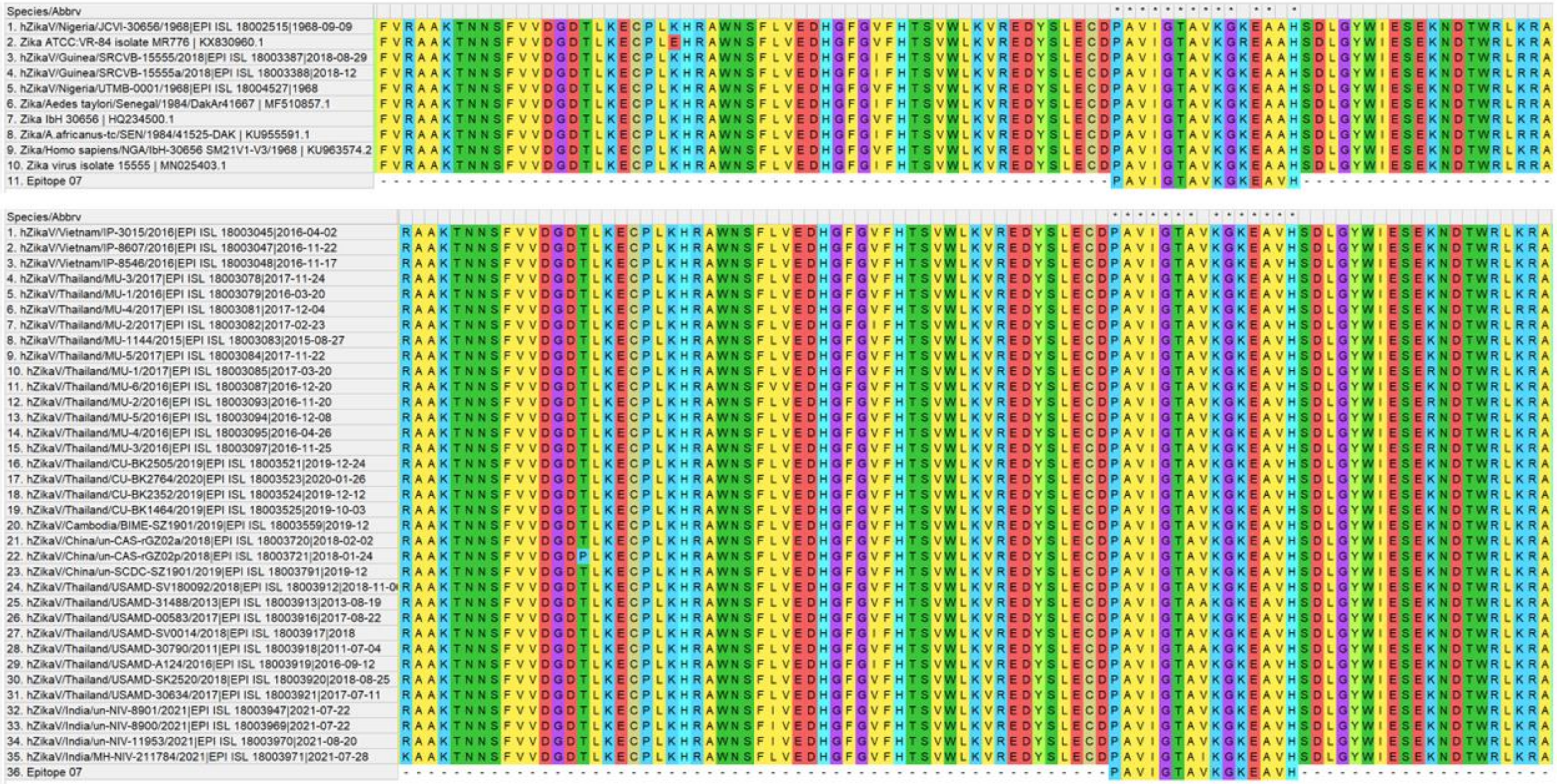
Species/Abbrv
1. hZikaV/Nigeria/JCVI-30656/1968|EPI ISL 18002515|1968-09-09
2. Zika ATCC:VR-84 isolate MR776 | KX830960.1
3. hZikaV/Guinea/SRCVB-15555/2018|EPI ISL 18003387|2018-08-29
4. hZikaV/Guinea/SRCVB-15555a/2018|EPI ISL 18003388|2018-12
5. hZikaV/Nigeria/UTMB-0001/1968|EPI ISL 18004527|1968
6. Zika/Aedes taylori/Senegal/1984/DakAr41667 | MF510857.1
7. Zika IbH 30656 | HQ234500.1
8. Zika/A.africanus-tc/SEN/1984/41525-DAK | KU955591.1
9. Zika/Homo sapiens/NGA/IbH-30656 SM21V1-V3/1968 | KU963574.2
10. Zika virus isolate 15555 | MN025403.1
11. Epitope 07
PAVIGTAVKGKEAVH
Species/Abbrv
1. hZikaV/Vietnam/IP-3015/2016|EPI ISL 18003045|2016-04-02
2. hZikaV/Vietnam/IP-8607/2016|EPI ISL 18003047|2016-11-22
3. hZikaV/Vietnam/IP-8546/2016|EPI ISL 18003048|2016-11-17
4. hZikaV/Thailand/MU-3/2017|EPI ISL 18003078|2017-11-24
5. hZikaV/Thailand/MU-1/2016|EPI ISL 18003079|2016-03-20
6. hZikaV/Thailand/MU-4/2017|EPI ISL 18003081|2017-12-04
7. hZikaV/Thailand/MU-2/2017|EPI ISL 18003082|2017-02-23
8. hZikaV/Thailand/MU-1144/2015|EPI ISL 18003083|2015-08-27
9. hZikaV/Thailand/MU-5/2017|EPI ISL 18003084|2017-11-22
10. hZikaV/Thailand/MU-1/2017|EPI ISL 18003085|2017-03-20
11. hZikaV/Thailand/MU-6/2016|EPI ISL 18003087|2016-12-20
12. hZikaV/Thailand/MU-2/2016|EPI ISL 18003093|2016-11-20
13. hZikaV/Thailand/MU-5/2016|EPI ISL 18003094|2016-12-08
14. hZikaV/Thailand/MU-4/2016|EPI ISL 18003095|2016-04-26
15. hZikaV/Thailand/MU-3/2016|EPI ISL 18003097|2016-11-25
16. hZikaV/Thailand/CU-BK2505/2019|EPI ISL 18003521|2019-12-24
17. hZikaV/Thailand/CU-BK2764/2020|EPI ISL 18003523|2020-01-26
18. hZikaV/Thailand/CU-BK2352/2019|EPI ISL 18003524|2019-12-12
19. hZikaV/Thailand/CU-BK1464/2019|EPI ISL 18003525|2019-10-03
20. hZikaV/Cambodia/BIME-SZ1901/2019|EPI ISL 18003559|2019-12
21. hZikaV/China/un-CAS-rGZ02a/2018|EPI ISL 18003720|2018-02-02
22. hZikaV/China/un-CAS-rGZ02p/2018|EPI ISL 18003721|2018-01-24
23. hZikaV/China/un-SCDC-SZ1901/2019|EPI ISL 18003791|2019-12
24. hZikaV/Thailand/USAMD-SV180092/2018|EPI ISL 18003912|2018-11-0
25. hZikaV/Thailand/USAMD-31488/2013|EPI ISL 18003913|2013-08-19
26. hZikaV/Thailand/USAMD-00583/2017|EPI ISL 18003916|2017-08-22
27. hZikaV/Thailand/USAMD-SV0014/2018|EPI ISL 18003917|2018
28. hZikaV/Thailand/USAMD-30790/2011|EPI ISL 18003918|2011-07-04
29. hZikaV/Thailand/USAMD-A124/2016|EPI ISL 18003919|2016-09-12
30. hZikaV/Thailand/USAMD-SK2520/2018|EPI ISL 18003920|2018-08-25
31. hZikaV/Thailand/USAMD-30634/2017|EPI ISL 18003921|2017-07-11
32. hZikaV/India/un-NIV-8901/2021|EPI ISL 18003947|2021-07-22
33. hZikaV/India/un-NIV-8900/2021|EPI ISL 18003969|2021-07-22
34. hZikaV/India/un-NIV-11953/2021|EPI ISL 18003970|2021-08-20
35. hZikaV/India/MH-NIV-211784/2021|EPI ISL 18003971|2021-07-28
36. Epitope 07
PAVIGTAVKGKEAVH
