# Supplementary material for: Design, development, and validation of multi-epitope proteins for serological diagnosis of Zika virus infections and discrimination from dengue virus seropositivity
Source: PLoS Negl Trop Dis. 2024 Apr 18;18(4):e0012100. doi: 10.1371/journal.pntd.0012100 (PMC11025737; doi:10.1371/journal.pntd.0012100)
Supplement: Attachment — Submitted filename: Response Letter to the editor Pereira et al 2023.pdf [file pntd.0012100.s003.pdf]

CT Vacinas

February 21st, 2024.

Please reply to:

Professor Flávio G da Fonseca
Centro de Tecnologia de Vacinas
Universidade Federal de Minas Gerais
31270-901 - Belo Horizonte - MG – BRAZIL
e-mail: fdafonseca@icb.ufmg.br

**RESPONSE LETTER**

Dear Dr. Michael Gaunt,

We were pleased to know that our manuscript entitled " "Design, development, and validation of multi-epitope proteins for serological diagnosis of Zika virus infections and discrimination from dengue virus seropositivity" has been evaluated as acceptable for publication at PLOS Neglected Tropical Diseases, subject to appropriate revision and response to the reviewers' comments.
We thank you and the reviewers for the positive assessment and the critical review. We carefully revised our manuscript and included a point-by-point response to the reviewers, and the modifications were highlighted in yellow in the resubmitted manuscript.

**AE COMMENTS**

**This is an impressive manuscript, very well written and well designed. There are two minor issues.**
**line 151-152**
**"For the construction of multi-epitope, recombinant proteins, known epitopes described in the literature and tested in serological assays were annotated." Can the authors rephrase this sentence because it isn't clear and an appropriate reference would be useful.**

**RESPONSE:** Thank you for the suggestion, the phrase was reformulated and four additional references were added. Furthermore, as suggested by Reviewer 3, a spreadsheet containing all epitopes was added to the supplementary material (Table S1).
"For the construction of multi-epitope proteins, B cell linear epitopes previously described in the literature and that had been tested in serological assays were annotated (18–21)." (lines 139-141)
**line 233-234**

**"Statistical analyses were performed using GraphPad Prism 9 program with the Two-way ANOVA test with multiple comparisons.**
**Its "two-way ANOVA" (small "t"). To avoid controversy - the authors have not checked whether the conditions of ANOVA are met, kindly add the following statement, "Non-parametric statistics provide an alternative approach to significance testing which could be explored in future research."**
**This is to provide cover for the authors should the Fmax test fail by a future investigator. If the authors have a strong Fmax value the above sentence can be ignored - providing the value is reported. Generally the key conditions for ANOVA have been met, i.e. they are truly independent and "homogeneity of variance" in the authors data looks about right. The ROC is the right test here so thats fine.**

**RESPONSE:** Thank you for the correction! It was very kind of you. The phrase was added to the manuscript. (lines 224-225)

**Reviewers comments**

**NOTE**: We are deeply thankful to the reviewers for their time, expertise and pertinent considerations.

**Reviewer 1 is requesting sera of "African or Asian origin". This will- be extremely difficult for the authors to obtain. One issue is Brazil has very strict biomaterial exportation laws and this makes reciprocation difficult, also the scarcity of this sera will make it hard. I agree bioinformatics analyse will address Reviewer 1's request, i.e. amino acid homology and predicted antigenicity. All other requests by the Reviewers are helpful for strengthening the manuscript and should be addressed.**

**RESPONSE:** We agree that analyzing samples from African and Asian countries would be very interesting for the development of our work. However, as pointed out by the Editor, the issue of importing such samples is quite difficult and would not be possible to complete in the available time window. We believe that bioinformatics analysis will greatly add to the manuscript. Once again, we thank the Editor for such kind and careful consideration.

**RESPONSES TO QUESTIONS RAISED BY THE REVIEWERS**

**REVIEWER #1:**

**This study describes the design and expression of recombinant proteins comprised of antibody epitopes specific for Zika virus, and predicted to not cross react with anti-dengue virus sera. Distinguishing between ZIKV and DENV (or other flaviviruses) in infected individuals is potentially quite significant, as it may influence subsequent medical interventions. The epitopes are within the NS1 protein, and have previously been described. Three recombinant proteins were expressed, containing 4 (ZIKV-1), 5 (ZIKV-2) or 6 NS1 epitopes divided by spacers of 5-amino acids. The proteins were used to coat ELISA plates and tested against sera from to cohorts of patients with confirmed ZIKV, DENV, or CHIKV infection. The smaller two recombinant proteins (ZIKV-1 and ZIKV-2) showed sensitivity and specificity that exceeded reported values for several commercially available tests.**
**In general, this is a well written manuscript that clearly shows the utility of the bacterially expressed multi-epitope proteins in distinguishing ZIKV infection from DENV, and likely other flavivirus infections. However, clarification of a few points would strengthen the overall impact.**

**1 - The cohorts used to test the sensitivity/ specificity are both of Brazilian origin, indicating infection with an Asian-American lineage ZIKV. Do the ZIKIV epitope recombinant proteins (or ZIKV-2, specifically) interact with antibodies from sera of African or Asian origin? If these cohorts are not available, could the authors can provide information about the conservation of the chosen epitopes in Asian or African ZIKV lineages?**

**RESPONSE:** We thank the reviewer for the observations and important suggestion. It was not possible to have access to samples from other countries. As mentioned by the editor-in-chief, Brazil has strict rules for importing biological material, and such importation would take too long considering the available time frame. With this, an epitope conservation analysis was carried out using bioinformatics, analyzing 35 strains of Asian origin and 10 strains of African origin. The analysis will enhance the work, thanks for suggestions. The paragraph below has been added to the text and the alignments have been added to the supplementary material (Figure S1).
"Furthermore, the epitopes present in the proteins show high conservation between strains of African and Asian origin. An analysis made with ten strains of African origin and thirty-five strains of Asiatic

Rua Professor José Vieira de Mendonça, 770 | sala 220
Bairro Engenho Nogueira | Belo Horizonte - MG
CEP 31310-260
(31) 3401.1113
www.ctvacinas.ufmg.br

origin shows that the Epitope 1 (HMCDATMSYECPMLDEGV) has 100% similarity with African strains and 100% with Asiatic strains. The Epitope 2 (HKKGEARRSRRAVTLPSH) has 100% similarity with African strains and only one of the Asian strains showed a difference in one amino acid when compared to the epitope sequence. The Epitope 3 (TVNMAEVRSYCYEASIS) has 88% similarity with the African and Asian strains. The Epitope 4 (TGVFVYNDVEAWRDRYKY) has a valine amino acid in position 5, five strains also have the valine amino acid while five other strains have the isoleucine amino acid, however, they have 100% similarity with the Asiatic strains. The Epitope 5 (SVEGELNAILEENGV) has 100% similarity with African strains and 100% similarity with thirty-one Asian strains and one amino acid difference with the other four strains. The Epitope 6 (WGKSYFVRAAKTNNSFVVDGDTLKECPLKH) has 100% similarity with nine African strains and presented a difference of one amino acid for one strain and 100% similarity with thirty-three Asian strains and presented a difference of one amino acid for two strains. The Epitope 7 (PAVIGTAVKGKEAVH) has 100% similarity with nine African strains and presented a difference of one amino acid for one strain and 100% similarity with thirty-two Asian strains and a difference of one amino acid with three strains (Supplementary material). This high conservation suggests that the multiepitope proteins can be used not only with Brazilian samples but also with samples from other countries." (lines 442-466).

**2 - Can the recombinant proteins be used to detect specific IgM responses? Because identification of the infecting virus may be of the most benefit as early in infection as possible, assessing the IgM response may increase the significance of this assay.**

**RESPONSE:** We thank the reviewer for the suggestion. We agree that early diagnosis is crucial, especially for the initial clinical management of the infection. Today we know that clinical management is not the same for all arboviruses. However, despite some attempts we were not able to access positive IgM samples. This is an important issue that should be further consideration during future developments. In order to point that out we added the following sentence to the text.
"Further developments of the diagnostic tool proposed here will include testing IgM positive samples for either Dengue or Zika, as IgM is an important marker for recent or ongoing infection " (lines 492 to 494).

**REVIEWER #3:**

**Overall, the study demonstrates a well-defined methodology, population description, and statistical analysis to address the stated objectives. However, explicit justification of sample size would help the manuscript. There are some concerns about the number of Chikungunya and RF+ samples used, but this is more in the context of next steps for the test developed not the manuscript. This is why a justification of the sample size at this stage is critical not necessarily an increase in sample size.**

**RESPONSE:** We thank the reviewer for the thoughtful consideration, to which we agree. The following phrase was added the manuscript.

"The number of samples used in our work was severely limited by sera availability. Due to the difficulties in correctly diagnosing arboviruses during epidemics in affected countries, the number of reliable samples we had access to is not so large. However, at this proof-of-principle stage, we believe the sample panel was sufficient to perform the tests and validations. Further validations of the proposed tests will surely require a larger sample size" (lines 409-414)

**The results are presented in a structured manner, with clear subsections describing different aspects of the analysis (e.g., protein modeling, purification, antigen evaluation). Figures and**

Rua Professor José Vieira de Mendonça, 770 | sala 220
Bairro Engenho Nogueira | Belo Horizonte - MG
CEP 31310-260
(31) 3401.1113
www.ctvacinas.ufmg.br

**tables are clear and are easy to read and understand. Visually they support the findings. Quantitative data, including sensitivity, specificity, and ROC curve analyses, are reported to quantify the performance of the diagnostic assays.**

**RESPONSE:** We thank the reviewer for this very positive assessment.

**The conclusions are supported by the data presented. For the most part the limitations of analysis are clearly designed. Any concerns on this point are highlighted in the summary. Public health relevance could be improved slightly by including a sentence on the level of ZIKV transmission currently.**

**RESPONSE:** We thank the reviewer for suggestion! A sentence with the current situation was added to the text.

"Currently, Brazil is facing the simultaneous circulation of Dengue, Zika and Chikungunya viruses, with a severe epidemic of Dengue leading to a high number of cases and deaths. Probable cases of Zika are currently low, considering the historical series; however, as of February 2024, 13 probable cases in pregnant women have been reported, two of which have been confirmed, showing the need for effective tests in the public health system (35). Nonetheless, it is worth noting that the system may be overloaded due to the dengue outbreak and therefore it is possible that not all samples are being tested for all arboviruses, and Zika cases may be underreported." (lines 369-376)

**Minor suggestions are included in the summary and general comments section.**

**Summary and General Comments**

**The study investigated the use of multi-epitope ZIKV proteins, where each epitope set 1, 2 and 3 include an increasing number of epitopes. The manuscript approach then utilizes AlphaFold for modeling and predicting structural variations. Comparative analysis revealed distinct structural differences, including sequence additions and intercalations, impacting potential binding sites and protein accessibility. Following protein expression and purification, antigenicity was evaluated using ELISA assays with ZIKV-positive, dengue virus (DENV)-positive, and negative sera samples. While ZIKV-1 and ZIKV-2 exhibited robust sensitivity and specificity in detecting ZIKV antibodies, ZIKV-3 showed lower performance, potentially attributed to its structural modifications. The results suggest the potential utility of certain multi-epitope proteins as effective antigens for ZIKV detection, emphasizing the importance of structural considerations in antigen design and evaluation for diagnostic purposes.**
**There are several minor concerns/weaknesses with the current manuscript. Overall, the study presents a promising approach to address the diagnostic challenges associated with ZIKV infection. At this point in the development of this test, it is best to address the following questions within the text. It may help further validation and consideration of potential limitations to ensure the reliability and clinical relevance of the developed diagnostic test in future work.**

**Minor concerns:**

**1. The cut-off's for the ELISA's as described in lines 221-226, What samples were used to determine the negative? Was it sample set 1 (FUNED) or sample set 2 (FAMERP)? Which protein was used ZIKV 1, 2 or 3?**

**RESPONSE:** We thank the reviewer for the inquiries**.** To determine the negatives, the set of samples provided by FAMERP were used. As described in the item "Human sera samples" lines 127-140, we only had negative sera provided by set 2. Furthermore, the three proteins (ZIKV-1, ZIKV-2 and ZIKV-3) were used, as cohort points were determined for each protein.

**What is the rationale for the use of Urea in one of the ELISA washes? Were pilot experiments completed without the Urea? And if yes what were the overarching results. In most common CLIA certified ELISA tests Urea is not commonly included in one of the wash steps.**

**RESPONSE:** We thank the reviewer for the inquiries**.** During the execution of the pilot experiments, we had problems with non-specific antibody binding, with high background noise. To correct this problem, we found that some authors had already used urea washing to reduce these non-specific bindings. The use of washing with 6 M urea favored an increase in the ZIKV+/DENV+ ratios. Tsai et al. (2018) described that washing with 6M urea for 5 minutes, at room temperature, after incubation with serum decreased the cross-reaction between ZIKV+ and DENV+ in an IgG ELISA assay. Wang et al. (2019) also used urea washing in ELISA to detect Treponema pallidum and managed to reduce false-positive results. Conzentino et al. (2022) used urea in the wash buffer to reduce background. Urea is a dissociating agent, so it can dissociate non-specific reactions, without harming specific reactions, since specific reactions have greater affinity than non-specific ones (BOBIĆ et al., 2009; WANG et al., 2019). Therefore, we added this wash after incubation with the sample and observed better results in reducing background noise.

TSAI, W.-Y. et al. Use of Urea Wash ELISA to Distinguish Zika and Dengue Virus Infections. **Emerging infectious diseases**, v. 24, n. 7, p. 1355–1359, jul. 2018.

WANG, Q. et al. Urea-mediated dissociation alleviate the false-positive Treponema pallidum-specific antibodies detected by ELISA. **PloS one**, v. 14, n. 3, p. e0212893, 2019.

CONZENTINO, M. S. et al. A magnetic bead immunoassay to detect high affinity human IgG reactive to SARS-CoV-2 Spike S1 RBD produced in Escherichia coli. **Brazilian journal of microbiology : [publication of the Brazilian Society for Microbiology]**, p. 1–7, abr. 2022.

BOBIĆ, B. et al. Comparative evaluation of three commercial Toxoplasma-specific IgG antibody avidity tests and significance in different clinical settings. **Journal of medical microbiology**, v. 58, n. Pt 3, p. 358–364, mar. 2009.

**The table of all original epitopes from the literature should be included as supplemental data or an external link included. Are the previous identified individual epitopes known or thought to be linear epitopes or are they conformational epitopes? The discussion should include future directions for this specific test.**

**RESPONSE:** We thank the reviewer for the observations and important suggestion. The table containing the epitopes was added in the supplemental dataset (Table S1). The selected epitopes are linear. The epitopes were predicted by the authors using linear B cell epitope predictor software. The information was added in the manuscript as shown bellow.

"For the construction of multi-epitope proteins, linear epitopes of B cell previously described in the literature and that had been tested in serological assays were annotated (18–21)." (line 139-141).

Sincerely yours,

Flavio G da Fonseca, PhD
